# Quantity and Quality of Carbohydrate Intake during Pregnancy, Newborn Body Fatness and Cardiac Autonomic Control: Conferred Cardiovascular Risk?

**DOI:** 10.3390/nu9121375

**Published:** 2017-12-19

**Authors:** Kirsty M. Mckenzie, Hasthi U. Dissanayake, Rowena McMullan, Ian D. Caterson, David S. Celermajer, Adrienne Gordon, Jonathan Hyett, Alice Meroni, Melinda Phang, Camille Raynes-Greenow, Jaimie W. Polson, Michael R. Skilton

**Affiliations:** 1 Boden Institute of Obesity, Nutrition, Exercise & Eating Disorders, D17-Charles Perkins Centre, University of Sydney, Camperdown, NSW 2006, Australia; kirstymacknz@gmail.com (K.M.M.); hdis1775@uni.sydney.edu.au (H.U.D.); rowenamcm@gmail.com (R.M.); ian.caterson@sydney.edu.au (I.D.C.); alice.meroni@sydney.edu.au (A.M.); melinda.phang@sydney.edu.au (M.P.); 2 Sydney Medical School, D17-Charles Perkins Centre, University of Sydney, Camperdown, NSW 2006, Australia; David.Celermajer@health.nsw.gov.au (D.S.C.); adrienne.gordon@sydney.edu.au (A.G.); Jon.Hyett@sswahs.nsw.gov.au (J.H.); jaimie.polson@sydney.edu.au (J.W.P.); 3 Royal Prince Alfred Hospital, Missenden Road, Camperdown, NSW 2050, Australia; 4 Sydney School of Public Health, Edward Ford Building, Fisher Road, University of Sydney, Sydney, NSW 2006, Australia; camille.raynes-greenow@sydney.edu.au; 5 School of Medical Science & Bosch Institute, Anderson Stuart Building (F13), University of Sydney, Sydney, NSW 2006, Australia

**Keywords:** carbohydrate, maternal diet, fibre, glycaemic index, glycaemic load, autonomic function, body composition, heart rate variability, infant, adiposity

## Abstract

The fetal environment has an important influence on health and disease over the life course. Maternal nutritional status during pregnancy is potentially a powerful contributor to the intrauterine environment, and may alter offspring physiology and later life cardio-metabolic risk. Putative early life markers of cardio-metabolic risk include newborn body fatness and cardiac autonomic control. We sought to determine whether maternal dietary carbohydrate quantity and/or quality during pregnancy are associated with newborn body composition and cardiac autonomic function. Maternal diet during pregnancy was assessed in 142 mother-infant pairs using a validated food frequency questionnaire. Infant adiposity and body composition were assessed at birth using air-displacement plethysmography. Cardiac autonomic function was assessed as heart rate variability. The quantity of carbohydrates consumed during pregnancy, as a percentage of total energy intake, was not associated with meaningful differences in offspring birth weight, adiposity or heart rate variability (*p* > 0.05). There was some evidence that maternal carbohydrate quality, specifically higher fibre and lower glycemic index, is associated with higher heart rate variability in the newborn offspring (*p* = 0.06). This suggests that poor maternal carbohydrate quality may be an important population-level inter-generational risk factor for later cardiac and hemodynamic risk of their offspring.

## 1. Introduction

Cardiovascular disease (CVD) remains the leading cause of morbidity and mortality worldwide. The World Health Organization estimates that 17.5 million people die each year from CVD, an estimated 31% of all deaths worldwide [1]. Both human and animal studies have provided strong evidence to suggest that the fetal environment impacts the risk of cardio-metabolic diseases in later life.

Impaired fetal growth is recognized as an important early life risk factor for CVD [2,3,4]. Fetal growth is affected by a range of genetic, environmental and maternal factors, and impaired fetal growth is associated with higher blood pressure and markers of poorer vascular health from early childhood through adulthood [5,6], which likely mediate at least part of the association with CVD. High birth weight is also an important risk factor for obesity in adulthood [7], and therefore also poses a risk for CVD. A proportion of the association between obesity and CVD may relate to autonomic dysfunction [8], an important factor in the early aetiology and ongoing pathophysiology of hypertensive and cardiovascular disorders [9,10,11,12,13]. Indeed, there is evidence that newborns with high body fatness, putatively a better marker of risk than birth weight, have altered autonomic control [14].

Maternal nutritional status is a powerful contributor to the overall intrauterine environment to which the fetus is exposed, with considerable evidence linking dietary restrictions to adverse offspring outcomes. For example, the offspring of women exposed to extreme famines while pregnant, such as the Dutch Hunger Winter, have reduced birth weights [15], subsequently linked to CVD and increased mortality in later life [16]. However, severe dietary restriction is uncommon in developed nations, in which dietary composition is likely to be of greater relevance. Both maternal dietary protein and fat intake have been shown to influence offspring cardiometabolic function, playing an integral role in the modification of genetic programming of fetal physiology and morphology in experimental models [17]. However, as glucose is the main energy substrate for fetal growth, maternal dietary carbohydrate intake is an important fetal exposure. Both dietary glycaemic index (GI) and glycaemic load (GL) are markers of dietary carbohydrate quality, and are strong determinants of circulating glucose levels throughout the day during pregnancy [18]. In turn, blood glucose levels are associated with increased risk of adverse outcomes in the offspring, including being born large-for-gestational age, in a linear dose-dependent manner [19].

Nonetheless, there is limited evidence directly linking maternal dietary carbohydrate quantity and quality with offspring body fatness and cardiac autonomic control during the perinatal period.

Therefore, we sought to determine whether maternal dietary carbohydrate intake, and GI and fibre as markers of the quality thereof, are associated with newborn anthropometric measures including body fatness, and cardiac autonomic function. We predicted that maternal intake of higher quality carbohydrates during pregnancy would be correlated with reduced body fatness and improved markers of infant autonomic control.

## 2. Materials and Methods

### 2.1. Participants

Mothers and their newborn babies were recruited (*n* = 224; between April 2015 and September 2016) from the maternity wards of RPA Women & Babies, Royal Prince Alfred Hospital, Sydney, Australia as part of a broader study examining the associations of body fatness, diet and late preterm birth with cardiovascular risk in the offspring. Participants were eligible if they met all of the following criteria: singleton born at the RPA Women & Babies, infants had body fatness assessed by air-displacement plethysmography within 24 h of birth, and gestational age ≥ 34 weeks. Exclusion criteria included infants with major congenital abnormalities or who required respiratory support, and those from a multiple birth pregnancy. For this manuscript, mothers with gestational diabetes mellitus (GDM) (*n* = 36), preeclampsia (*n* = 8), and neonates born pre-term (gestational age ≤ 36 weeks, *n* = 8), were excluded prior to analysis, ultimately leaving 142 participants for analysis. 

The study was conducted in accordance with ethical standards and approved by the Sydney Local Health District (HREC/14/RPAH/478). Participation was voluntary and informed written consent was obtained from the participating mothers before taking part in the study.

### 2.2. Maternal Characteristics and Dietary Intake

A self-administered questionnaire recorded maternal demographic characteristics and perinatal outcomes, with confirmation from health records.

Dietary data and intakes during pregnancy were collected using a validated electronic food frequency questionnaire (FFQ) which includes the foods and drinks typically consumed in Australia [20]. Women were requested by the study investigators to specifically think about their dietary consumption during the entirety of their pregnancy when completing the FFQ. Food specific nutrient values were derived from AUSNUT 2007, and GI and GL values from NUTTAB 2010. 

### 2.3. Infant Anthropometry and Autonomic Function

Birth weight, length and head circumference were collected clinically as part of the newborn early assessment program (RPAH_GL2013_019) and obtained from medical records. Infant body fat percentage, lean body mass and body composition were assessed by air-displacement plethysmography (PEA POD, COSMED, Italy) as part of routine clinical practice [21]. Gestational age was estimated from the date of last menstrual period and early pregnancy ultrasound [22].

Autonomic function was assessed as heart rate variability (HRV) derived from electrocardiogram (ECG) recordings. ECG was recorded continuously for 15 min using a standard neonatal 3-lead configuration (Powerlab, ADInstruments, Australia) in infants sleeping in a supine position. Analog output was digitized at 500 Hz. During recordings, the infants’ behavior were closely monitored, noting any occasions when the infant woke up, which were subsequently removed from analysis. Frequency domain analysis of HRV was performed using LabChart (HRV 1 module, version 7, ADInstruments, Australia) from 3 R-R interval epochs of exactly 4 min. Peak detection on the ECG signal was used to create RR interval sequences. A fast Fourier transformation (256 point, Hanning window) with 50% overlap was used to analyze the frequency domain. A range of 0–1.1 Hz was used to investigate the spectral bands for HRV that incorporated the respiratory frequency of the infant [23]. High frequency (HF) was defined at 0.15 to 1.1 Hz and low frequency (LF) at 0.04 to 0.15. The very low frequency band was not analyzed because of probable non-harmonic components and trend removal artifacts [24]. Of the 142 participants who met the inclusion criteria for analysis, successful cardiac autonomic function assessments were available for 101 infants. The primary reasons for unsuccessful cardiac autonomic function assessment were infants failing to settle during the recordings, early discharge prior to assessment and equipment related issues. 

### 2.4. Statistical Analysis

Descriptive data are presented as mean (SD) for continuous variables and *n* (%) for categorical variables, unless otherwise stated. Data were visually assessed for normality and confirmed using the Kolmogorov-Smirnov test. Non-normally distributed data were transformed using an appropriate transformation. Associations between maternal dietary factors, newborn autonomic functions, and body composition measures were determined by multivariable linear regression models, adjusting for maternal total energy intake during pregnancy, maternal age and newborn gender as covariates. Our sample size of *n* = 142 mother-child pairs provides us with 85% power to detect a correlation coefficient of 0.25 at *2p* < 0.05. 

Maternal carbohydrate intake during pregnancy was derived from the FFQ as crude intake (g/day), and subsequently converted to proportion of daily energy intake (%) for statistical analysis using a conversion factor of 17 kJ per gram of carbohydrate [25]. Maternal dietary intakes, specifically carbohydrate intake, GI, GL and fibre were analyzed as continuous variables and as categorical variables derived from quartiles. Cut-offs for quartiles were: Carbohydrate: Min 28.6% of total energy intake; 25th percentile 37.0; 50th percentile 40.9; 75th percentile 43.3; max 59.1. GI: Min 41.7% of total energy intake; 25th percentile 47.0; 50th percentile 49.8; 75th percentile 52.1; max 59.8.Quartiles for fibre (g/day) and GL were derived using the residual method [26]. For the four groups stratified by carbohydrate intake, the mean macronutrient proportions (Carbohydrate:Fat:Protein) were: Q1 34:42:22; Q2 39:40:19; Q3 42:37:19; Q4 47:33:18. All statistical analysis was undertaken with IBM SPSS Statistics (version 22.0; IBM Corp., Somers, NY, USA), and statistical significance inferred at *2p* < 0.05.

## 3. Results

### 3.1. Demographics

Characteristics of the 142 mother-child pairs are shown in Table 1. Mothers who participated in the study were, on average, in their early thirties (33 years (SD 4.4)) and with pre-pregnancy BMI (22.0 (IQR 3.6) kg/m^2^). On average, women obtained 41% of their daily energy intake from carbohydrate, GI was relatively low and fibre intake was slightly lower than the recommended adequate intake for pregnant women in Australia [27] (Table 1).

### 3.2. Body Composition and Maternal Carbohydrate Intake

There was no association of carbohydrate intake, either quantity or quality, with infant birth weight or body fatness (Table 2). Results were similar when dietary exposures were treated as continuous or categorical variables. Further adjustment for carbohydrate intake did not modify the association of maternal dietary fibre intake with newborn body fat or birth weight.

### 3.3. Infant Autonomic Function and Maternal Carbohydrate Intake

**Figure 1:**
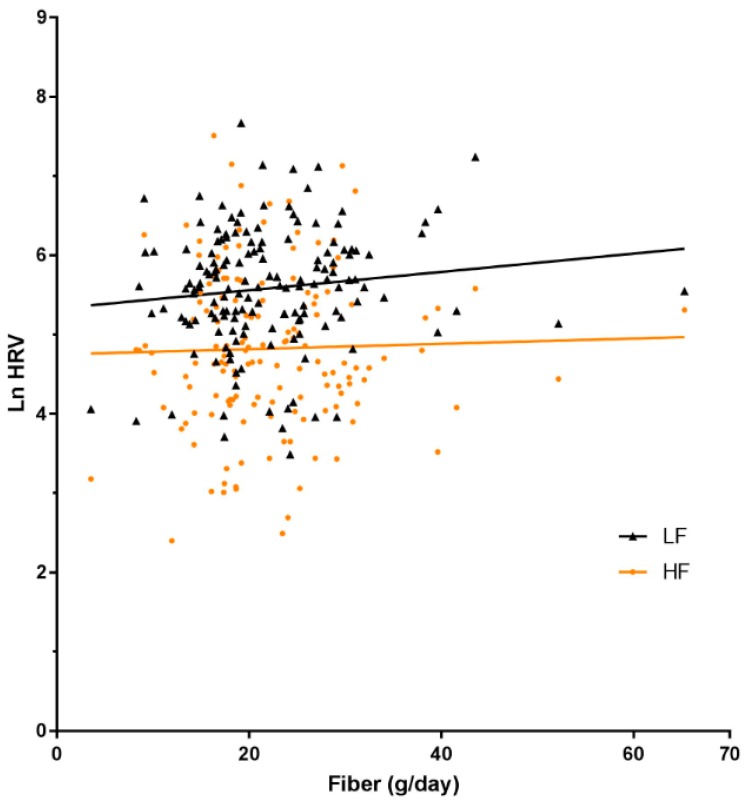
Correlation between maternal fibre (g/day) intake for the duration of pregnancy and newborn autonomic function (HRV).

In the 101 infants in whom cardiac autonomic control was assessed, maternal carbohydrate intake as a proportion of energy was not associated with measures of cardiac autonomic control (total power, LF, HF and LF:HF). There was weak evidence of an association of better carbohydrate quality (higher fibre, lower GI) with higher LF band of heart rate variability, considered to be a marker of sympathetic modulation. This was similar for analysis of GI as either a continuous variable or as a categorical variable (Table 3), and for fibre (Table 3; Figure 1). These associations were slightly strengthened by adjustment for pre-pregnancy weight, e.g., LF: −2.17 [−4.36, 0.03] per unit GI (*p* = 0.05) and 0.66 (0.08, 1.24) per unit fibre (*p* = 0.03) and were only slightly weakened by adjustment for newborn body fatness, e.g., LF: (−1.8 [−4.0, 0.4]) per unit GI (*p* = 0.12), adjusted for body fatness (%).

## 4. Discussion

Our findings indicate maternal carbohydrate quality, as measured by GI and fibre intake, are weakly associated with physiological differences in infant cardiac autonomic function. In contrast, the quantity of maternal carbohydrate intake during pregnancy was not associated with meaningful differences in offspring body composition or autonomic control, including amongst those who consumed the highest proportion of energy as carbohydrate. Autonomic nervous system activity predicts future risk of cardiovascular disease [24,28] and plays a role in the development and etiology of adult hypertension [29,30]. HRV is a non-invasive marker of autonomic modulation of heart rate and is categorised into three main spectral components; the frequency domain measures of very low frequency (VLF), low frequency (LF) and high frequency (HF). Both the distribution of the measure of total power, as well as the power within the LF and HF bands may vary in relation to changes in autonomic modulations of heart rate [31,32]. The HF band is associated with respiratory sinus arrhythmia and is largely influenced by vagal nerve activity, while the interpretation of the LF band is more complex [24]. The LF component is believed to reflect both vagal and sympathetic activity [33,34], or alternatively as a quantitative marker of sympathetic modulations [35,36]. Accordingly our finding that carbohydrate quality was weakly associated with the LF band of heart rate variability, suggests that higher quality maternal carbohydrate intake may be linked with improved sympathetic modulation in their newborn offspring. Conversely, the offspring of women who consumed poorer quality carbohydrates during pregnancy may have worse cardiac autonomic control, which may mechanistically predispose these infants to later hemodynamic and cardiovascular disorders. Future studies may determine whether this is indeed the case, and if so, seek to quantify the effectiveness of interventions that seek to improve maternal carbohydrate quality during pregnancy [22].

The quality of maternal carbohydrate intake during pregnancy influences fetal glucose supply and consequently fetal growth [19,37,38]. The adverse newborn cardio-metabolic profile associated with higher maternal glucose levels may therefore result from a complex interaction between fetal growth, body fatness, vascular changes and cardiac autonomic modulation, which is consistent with the association between autonomic function and maternal carbohydrate quality being weakened by adjustment for newborn birth weight or body fatness.

Carbohydrates represent the largest individual macronutrient component of most diets [39], and both their quantity and quality has been independently associated with maternal blood glucose concentrations and pregnancy outcomes [40]. In this study we used GI and fibre as measures of carbohydrate quality. Both are components of healthy dietary patterns and are associated with improved cardio-metabolic profiles in adults [41,42,43]. While strong continuous associations have been found between maternal glucose levels and the incidence of infant macrosomia [19,40], low GI dietary interventions in pregnant women have not been clearly demonstrated to affect the incidence of macrosomia, and have little influence on birth weight [40,44,45] or body composition in infancy [46]. We saw no clear or consistent evidence for an association between maternal dietary carbohydrate quality and newborn birth weight or body fatness. This may be explained by the robust physiological transfer of nutrients from mothers to the developing fetus, within the normal range of dietary intakes of women with uncomplicated pregnancies living in developed nations. Indeed, the body fatness and fat free mass of newborns in our study were similar to healthy values from normative data [47]. However, our findings differ from those of a previous small study which showed an inverse association between dietary GI and infant fat free mass in a group of women at risk of GDM [48]. These differences may relate to the risk nature of the participants or timing of dietary assessment.

In contrast, there is evidence that interventions that target GI may be associated with improved vascular health outcomes in the offspring during infancy [22]. This complements our finding that carbohydrate quality may be weakly associated with cardiac autonomic control in newborns. Our results expand upon the etiology of early vascular disease and haemodynamic risk, and inform on age-appropriate cardiovascular risk markers that may identify risk relating to maternal dietary intake. It remains to be determined whether the effect size of the observed association of carbohydrate quality with cardiac autonomic control would be more marked in pregnancies at high risk of poorer cardio-metabolic outcomes.

The main strength of this study lies in the use of air-displacement plethysmography to assess newborn body fatness and fat free mass [49]. There are relatively few studies of body fatness in newborns using air displacement plethysmography, which is the gold-standard non-invasive methodology. Newborn body fatness is potentially a more appropriate marker of the net effect of the intrauterine environment and the maternal nutritional state on fetal growth. Accordingly, it has been proposed that infant body fatness may be a more accurate predictor for later life CVD and metabolic disease, in comparison to newborn birth weight and lean body mass, which are known to be more greatly influenced by genetic factors [50]. Additionally, the intensive newborn measures collected, combined with the assessment of maternal [51] dietary intake, provide unique data regarding the potential associations between maternal dietary carbohydrate intake, infant autonomic function, and adiposity.

All dietary assessment methods have limitations, with FFQs being prone to mis- or under-reporting of exact quantities of nutrient intakes [52]. Accordingly, we have used measures that are proportionate to total energy intake (e.g., carbohydrate intake as percentage energy), and/or adjusted for total energy intake in our statistical analyses. The advantage of utilising a FFQ is that it removes the incentive for study participants to prospectively alter dietary intake, is less burdensome on the subjects [53], and provides a validated measure of habitual dietary intake. Assessment of dietary intake at multiple times during pregnancy, specifically during each trimester and also during the peri-conception period, would provide a clearer and more definitive indication of any critical timings of dietary intake during pregnancy. Blood pressure changes markedly during the early neonatal period, and is logistically difficult to obtain reproducible measures. Accordingly, we did not measure blood pressure in these participants, although it would have informed the cardiac autonomic function results. We excluded pregnancies affected by GDM and preeclampsia to avoid reverse causation, and as such our results should be taken within the context of healthy pregnancies. Future studies that prospectively assess diet in early pregnancy, prior to the development of GDM or preeclampsia, will inform further on whether these complications of pregnancy alter the observed associations. Our sample size is similar to other papers in the field detailing cardiac autonomic control in infants [54], but made sub-analysis of low birth weight babies unfeasible. Family history of cardiovascular disease and gestational weight gain also influence early life markers of cardiometabolic disease [55,56], although were not assessed in the current study. Our sample size also restricts our ability to study the interaction between maternal dietary intake and the genetic programming of cardiometabolic disease [17]. The role of these factors as putative confounders or mediators of the observed associations should be the focus of future studies with a larger sample size.

Weight trajectory during infancy and early childhood may also be an important predictor of later life cardiometabolic disease [5,6]. Future longitudinal studies taking into account the distinct associations of maternal carbohydrate quality and childhood growth trajectories with autonomic activity, would further inform on the independence and potential interaction between these early life exposures. 

## 5. Conclusions

Our findings suggest that quality, but not quantity, of carbohydrates consumed during pregnancy is weakly associated with cardiac autonomic control in the newborn offspring, highlighting a novel putative link between maternal dietary intake and offspring cardiovascular and haemodynamic risk. Whether these associations with cardiac autonomic control have potential long-term implications for disease outcomes, or in risk stratification, remains to be determined. Furthermore, our findings are consistent with a proportionally high carbohydrate diet having neither beneficial nor detrimental effects on newborn cardiovascular risk, birth weight and body fatness, in this otherwise healthy population.

## Figures and Tables

**Table 1:** Maternal characteristics, including diet, and neonatal anthropometry.

Characteristic		
*Maternalcharacteristics*		*Cardiac autonomic function subgroup*
Maternal age, years	33.3 ± 4.4	33.2 ± 4.6
Maternal height, m	165.3 ± 6.5	165.0 ± 6.6
Pre-pregnancy BMI, kg/m^2^	22.0 ± 3.6	22.1 ± 4.1
Total energy intake, kJ/day	7854 ± 3756	7478 ± 3464
Total fat intake, % energy	37.7 ± 4.6	37.9 ± 4.3
Protein intake, % energy	19.4 ± 2.8	19.0 ± 3.3
Carbohydrate, % energy	41.0 ± 5.0	41.0 ± 4.8
Fibre, g/day	22.8 ± 9.8	21.9 ± 8.7
Glycaemic Index	49.8 ± 5.1	49.4 ± 0.
Glycaemic Load	89.3 ± 44.8	96.3 ± 5.3
*Newborncharacteristics*		
Female, *n*%	83 (58%)	58 (57%)
Gestation, week	39.3 ± 1.2	39.2 ± 1.1
Birth Weight, g	3426 ± 521	3436 ± 558
Birth length, cm	49.9 ± 2.3	49.9 ± 2.5
Head circumference, cm	34.8 ± 1.4	34.8 ± 1.4
Body Fatness, %	11.1 ± 5.1	11.3 ± 5.4
Fat Free Mass, %	88.9 ± 5.1	88.7 ± 5.4
HRV – TP, ms^2^	-	1124 ± 1113
HRV – LF, ms^2^	-	285 ± 286
HRV – HF, ms^2^	-	122 ± 168

Values are mean ± SD for continuous variables and *n* (%) for categorical variables. *n* = 131 for maternal height, *n* = 134 for pre-pregnancy BMI, *n* = 147 for gestational age, birth weight, birth length, head circumference, body fatness and fat free mass. For the autonomic function subgroup, *n* = 91 for pre-pregnancy BMI, *n* = 104 for TP, LF & HF, *n* = 142 for all other variables and *n* = 101 for all other cardiac autonomic function variables. Pre-pregnancy BMI, GI, GL, TP, LF and HF were not normally distributed, and are expressed as median ± interquartile range (IQR).

**Table 2:** Associations between maternal carbohydrate intake, both quantity and quality, with newborn body composition (*n* = 142).

	Birth Weight (g)		Body Fatness (%)	
	*β* (95% CI)	*p* Value	*β* (95% CI)	*p* Value
Carbohydrate (%)	43 (−23, 108)	0.20	0.01 (−0.18, 0.16)	0.90
Q_1_	*Reference*		*Reference*	
Q_2_	222 (−33, 478)	0.09	2.18 (−0.30, 4.67)	0.09
Q_3_	143 (−109, 396)	0.26	0.35 (−2.11, 2.80)	0.78
Q_4_	124 (−124, 372)	0.33	1.44 (−0.98, 3.85)	0.24
Glycaemic Index	291 (−970, 1553)	0.65	1.73 (−10.59, 14.04)	0.78
Q_1_	*Reference*		*Reference*	
Q_2_	−13 (−259, 233)	0.91	−0.58 (−3.00, 1.83)	0.63
Q_3_	122 (−129, 372)	0.34	0.37 (−2.08, 2.82)	0.77
Q_4_	32 (−218, 282)	0.80	0.26 (−2.19, 2.71)	0.83
Glycaemic Load	265 (−138, 667)	0.20	0.88 (−3.07, 4.83)	0.66
Q_1_	*Reference*		*Reference*	
Q_2_	−236 (−478, 6)	0.06	−1.79 (−4.20, 0.61)	0.14
Q_3_	125 (−114, 363)	0.30	0.55 (−1.82, 2.93)	0.65
Q_4_	−56 (−294, 181)	0.64	−0.28 (−2.64, 2.08)	0.81
Fibre (g)	45 (−51, 141)	0.36	0.01 (−0.15, 0.16)	0.95
Q_1_	*Reference*		*Reference*	
Q_2_	−98 (−340, 144)	0.43	−0.11 (−2.42, 2.20)	0.93
Q_3_	97 (−147, 341)	0.43	2.56 (0.23, 4.90)	0.03
Q_4_	−30 (−273, 214)	0.81	−0.39 (−2.72, 1.94)	0.75

Values are unstandardized *β*-regression coefficients (95% CI) from multivariable regression analyses, and represent the differences in newborn birth weight (g) and body fatness (%) per unit increase in the independent variable (maternal dietary characteristic), adjusted for maternal age, newborn gender and total energy intake. Glycaemic Index and glycaemic load were log transformed (ln) for analysis.

**Table 3:** Associations between maternal carbohydrate content and quality with newborn heart rate variability (autonomic cardiac control).

	TP *, ms^2^	LF *, ms^2^			HF *, ms^2^		LF:HF *	
	* β* (95% CI)	* p* Value	* β* (95% CI)	* p* Value	* β* (95% CI)	* p* Value	* β* (95% CI)	* p* Value
Carbohydrate, %	0.01 (−0.04, 0.03)	0.75	0.00 (−0.03, 0.03)	0.97	0.00 (−0.04, 0.05)	0.96	0.00 (−0.03, 0.03)	0.85
Q_1_	*Reference*		*Reference*		*Reference*		*Reference*	
Q_2_	−0.03 (−0.50, 0.44)	0.90	−0.20 (−0.70, 0.30)	0.42	−0.32 (−0.96, 0.32)	0.32	0.23 (−0.22, 0.68)	0.31
Q_3_	0.17 (0.26, 0.60)	0.44	−0.11 (−0.34, 0.57)	0.62	0.22 (−0.37, 0.81)	0.46	−0.01 (−0.42, 0.40)	0.96
Q_4_	−0.03 (−0.46, 0.41)	0.91	−0.01 (−0.47, 0.45)	0.97	−0.09 (−0.67, 0.50)	0.76	0.12 (−0.29, 0.53)	0.57
Glycaemic Index *	−1.37 (−3.43, 0.70)	0.19	−1.94 (−4.12, 0.24)	0.08	−1.59 (−4.46, 1.27)	0.27	−0.10 (−2.09, 1.89)	0.92
Q_1_	*Reference*		*Reference*		*Reference*		*Reference*	
Q_2_	−0.11 (−0.51, 0.30)	0.61	−0.21 (−0.64, 0.21)	0.32	−0.28 (−0.84, 0.27)	0.32	0.10 (−0.29, 0.49)	0.60
Q_3_	0.00 (−0.46, 0.46)	0.99	−0.13 (−0.62, 0.35)	0.58	0.00 (−0.64, 0.63)	1.00	0.07 (−0.37, 0.51)	0.76
Q_4_	−0.31 (−0.73, 0.12)	0.16	−0.44 (−0.89, 0.01)	0.05	−0.42 (−1.01, 0.17)	0.16	0.06 (−0.35, 0.47)	0.77
Glycaemic load *	0.38 (−0.34, 1.09)	0.30	0.19 (−0.57, 0.95)	0.62	0.34 (−0.65, 1.33)	0.49	−0.15 (−0.83, 0.54)	0.67
Q_1_	*Reference*		*Reference*		*Reference*		*Reference*	
Q_2_	0.19 (−0.32, 0.62)	0.39	0.20 (−0.25, 0.65)	0.38	0.22 (−0.37, 0.82)	0.46	0.08 (−0.34, 0.49)	0.71
Q_3_	−0.05 (−0.48, 0.37)	0.80	−0.21 (−0.66, 0.23)	0.34	−0.11 (−0.69, 0.47)	0.71	0.05 (−0.36, 0.45)	0.82
Q_4_	−0.02 (−0.46, 0.42)	0.92	0.00 (−0.47, 0.46)	1.00	−0.15 (−0.76, 0.46)	0.62	0.14 (−0.29, 0.56)	0.52
Fibre, g	0.02 (−0.01, 0.05)	0.14	0.03 (−0.00, 0.06)	0.06	0.01 (−0.04, 0.05)	0.79	0.03 (−0.00, 0.06)	0.06
Q_1_	*Reference*		*Reference*		*Reference*		*Reference*	
Q_2_	0.23 (−0.19, 0.65)	0.29	0.45 (−0.01, 0.89)	0.05	0.28 (−0.31, 0.86)	0.35	0.19 (−0.21, 0.59)	0.34
Q_3_	0.24 (−0.19, 0.66)	0.28	0.21 (−0.24, 0.65)	0.36	0.09 (−0.51, 0.68)	0.78	0.26 (−0.15, 0.66)	0.22
Q_4_	0.27 (−0.16, 0.70)	0.22	0.40 (−0.05, 0.85)	0.08	0.15 (−0.45, 0.75)	0.62	0.26 (−0.15, 0.67)	0.21

Values are unstandardized *β*-regression coefficients (95% CI) from multivariable models (*n* = 101). Results shown are differences in newborn cardiac autonomic control per unit increase in maternal GI (glycaemic index), GL (glycaemic load) and carbohydrate intake (%), adjusted for maternal age, newborn gender and total energy intake. * Log transformed for analysis. TP, total power; LF, low frequency; HF, high frequency; LF:HF, ratio of low frequency to high frequency.
